# Estimating the potential public health impact of fibre enrichment: a UK modelling study

**DOI:** 10.1017/S0007114521004827

**Published:** 2022-11-14

**Authors:** Kirstie Canene-Adams, Ieva Laurie, Kavita Karnik, Brian Flynn, William Goodwin, Sandrine Pigat

**Affiliations:** 1 Tate & Lyle PLC, London, UK; 2 Creme Global, Dublin, Ireland

**Keywords:** Dietary modelling, Fibre, Reformulation, Enrichment

## Abstract

For improving human health, reformulation can be a tool as it allows individuals to consume products of choice while reducing intake of less desirable nutrients, such as sugars and fats, and potentially increasing intake of beneficial nutrients such as fibre. The potential effects of reformulating foods with increased fibre on diet and health need to be better understood. The objective of this statistical modelling study was to understand how fibre enrichment can affect the diet and health of consumers. The UK National Diet and Nutrition Survey datasets from 2014 to 2015 and 2015 to 2016 were utilised to evaluate intakes of fibre and kilocalories with a dietary intake model. Foods and beverages eligible for fibre enrichment were identified (*n* 915) based on EU legislation for fibre content claims. Those people who meet dietary reference values and fibre enrichment health outcomes such as weight, CVD and type 2 diabetes risk reductions were quantified pre- and post-fibre reformulation via Reynolds *et al.*, D’Agostino *et al.* and QDiabetes algorithms, respectively. The fibre enrichment intervention showed a mean fibre intake of 19·9 g/d in the UK, signifying a 2·2 g/d increase from baseline. Modelling suggested that 5·9 % of subjects could achieve a weight reduction, 72·2 % a reduction in cardiovascular risk and 71·7 % a reduced risk of type 2 diabetes with fibre fortification (all *P*s ≤ 0·05). This study gives a good overview of the potential public health benefits of reformulating food products using a straightforward enrichment scenario.

Reformulation refers to the process of altering foods and beverages to have changed nutritional composition between two time points to reduce levels of energy, total fat, saturated fat, Na and/or sugar^(1)^. Although reformulation typically focuses on reformulating these undesirable nutrients out of food, simultaneously enriching foods with desirable nutrients such as fibre can complement this reduction by means of displacing higher energy components of the food, thus making the food lower in energy content. There are different reasons for the reformulation of foods including the reduction of nutrients that are commonly consumed beyond the recommended intakes, such as sugar or Na, or the fortification of foods with more health-positive nutrients that may have been lost in processing or may not have been present to begin with. Additionally, reformulation can improve diets by increasing lacking nutrients in the current food supply, as is the case with fibre. Ultimately, reformulation is carried out with the aim of improving the nutritional profile of food and having a positive impact on the health of the consumer. There are also commercial benefits of reformulation such as claims, marketing to a new audience of consumers, increased sales by rejuvenating or extending a product line, or price increases due to premium ingredient additions.

A series of systematic reviews and meta-analyses consisting of millions of person years and over 200 prospective studies and clinical trials evaluated the scientific evidence among carbohydrate quality and non-communicable disease outcomes such as body weight, cholesterol and blood pressure^(2)^. Based on the association of reduced mortality risk, incidence of non-communicable diseases and risk factors, Reynolds *et al.* recommend at least 25–29 g of dietary fibre to be consumed a day which aligns with the WHO recommendations. Furthermore, their dose–response analysis suggested that higher intakes of dietary fibre could accrue even greater benefit to protect against CVD, type 2 diabetes, and colorectal and breast cancer. Reynolds *et al.* mention in their publication that much of the literature which contributed to their systematic review and meta-analysis was before the era of adding *Codex Alimentarius*-approved synthesised or extracted fibres that were commonly added to products globally. Since the EU Commission Directive 2008/100/EC Annex II, the U.S. Food and Drug Administration (FDA) in 21CFR §101.9 (e) (6) (i), *Codex Alimentarius* and other regulatory authorities approved fibres based on health benefits, and it is assumed in this publication’s modelling that human health benefits will extend to these novel synthesised or extracted fibres.

Yet, across the globe, average daily fibre intakes are well below the recommended amount. In the USA, mean fibre intake for adults is around a mere 16·1 g/d^(3)^ and in the UK fibre intake has been reported to be around 19 g/d^(4)^. There is clearly a need for increasing fibre intake for public health with reformulation being one of the ways to achieve this. While reformulating foods and the resulting possible health impacts are of interest for public health, this work needs to be better modelled and researched.

The objective of this study was to conduct a statistical modelling study to understand how fibre enrichment can impact the diet and health status of consumers. To achieve this, the UK National Diet and Nutrition Survey (NDNS)^(4)^ rolling programme, a UK food consumption survey, was used to create scenarios to model the dietary and health impacts of fibre reformulation. A dietary exposure model built by Creme Global was used to model these changes^(5)^. The food categories of interest for fibre enrichment in this study were identified as bakery products, beverages, dairy and dairy alternatives, soups, sauces and dressings and confectionery. Using these selected food categories, nutritional composition changes were implemented and their nutritional intake outcomes were assessed. Comparisons were made against the baseline diet (representing the market pre-reformulation) and the results were measured for their impact on public health outcomes.

## Methods

### Data source

The NDNS is a continuous, cross-sectional survey designed to collect detailed, quantitative information on the food consumption, nutrient intake and nutritional status of the general population aged 1·5 years and over living in private households in the UK^(4)^. This study used the most recently published data from the 7th and 8th year of the survey, carried out in 2014–2015 and 2015–2016, respectively. It was decided to use these 2 years rather than the full 8 years of the NDNS rolling programme, in order to reflect the most current consumption habits. This study used the entire cohort of 2723 subjects aged 1·5–94 years (inclusive) that had statistical weightings. Statistical weights or sampling weights indicate that an observation in a survey represents a certain number of people in a finite population, the UK population in the case of NDNS. The results of the Creme Food Data Science model and all summary statistics generated from health outcome modelling are statistically weighted. When running dietary assessments using the probabilistic model, a subset of the cohort of 2723 subjects is created in each scenario, representing ‘Consumers Only’ of the relevant food group.

### Food diaries, nutritional composition and recategorisation

Four-day food diaries are available for every subject in the NDNS which listed foods and beverages by name and associated quantity consumed. The nutritional information for each food and beverage consumed was available through the nutrients file provided with the NDNS datasets. Food Groups available in the NDNS years 7 and 8 (2014/2015–2015/2016) were regrouped into the following food groups: bakery, beverages, dairy and dairy alternatives, soups, sauces and dressings and confectionery. Where an Original Food Group in the NDNS was deemed to not match with any of these food groups, the Original Food Group in the NDNS was listed as ‘Other’. Within the Original Food Group in the NDNS called ‘MISCELLANEOUS’, foods/beverages were individually assessed based on their Food Names and placed in Food Groups or ‘Other’.

### Baseline intakes

A baseline assessment was run on Expert Models Food Data Science, a validated dietary intake model^(5,6)^ using the NDNS Dietary Survey (subject information, food diary information and nutritional information of the foods, specifically the nutrient values per 100 g of food/beverage of AOAC fibre and per 418·4 kJ [100 kcal], energy in kilocalories) as model inputs.

Nutrient intake was calculated as follows:

(Weight of food/beverage (g) consumed in a given eating event according to NDNS) × (Concentration of nutrient in that food or beverage (g/100 g) according to NDNS)

The nutrient intakes from each eating event were summed up per person per day, per nutrient. These values were divided by four to get an average daily nutrient intake over the 4-d survey period per person. Weighted intake statistics were then calculated for each population age group.

### Intervention intakes

#### Eligibility and calculation of altered concentration: fibre

The commercially prepared foods and beverages eligible for fibre enrichment were identified, and some exclusions were applied, such as chocolate confectionery due to composition legislation^(7)^ (Table 1). A total of 915 food and beverages were deemed eligible for fibre enrichment. The EU legislation for nutrition claims^(8)^ was considered in the concentration of fibre to be used at intervention. There were three scenarios for fibre intervention, for a food/beverage containing 0 g fibre/100 g the concentration was left at zero, others were ‘source of fibre’ and some enriched at a ‘high-fibre’ level. In order for a food or beverage to be labelled as a ‘source of fibre’, it must contain at least 3 g/100 g or 1·5 g/100 kcal. Therefore, for a food containing less than 3 g fibre/100 g, the concentration of fibre was brought to 3 g/100 g or a beverage was brought to 1·5 g/100 kcal. For a food containing greater than or equal to 3 g fibre/100 g, 3 g of fibre was added. The rationale behind the addition of 3 g to these foods rather than a higher or lower value was based on the EU legislation for nutrition claims^(9)^. For a food to be labelled ‘high fibre’, it must contain at least 6 g fibre/100 g. As a conservative approach, 3 g of fibre was added to the foods containing greater than or equal to 3 g/100 g already. Foods that were not eligible for inclusion in the reformulation scenario for reasons listed above were still included in the calculation of dietary intakes of fibre for the total population. Therefore, the baseline and intervention scenarios modelling fibre intake include these foods as well as those that had been enriched with additional fibre. It should be noted that the foods and beverages deemed eligible for fibre enrichment were commercially prepared foods for which consumption levels already existed, and thus the study did not model the creation of new processed foods.

**Table 1.** Foods included or excluded in the fibre enrichment intervention

Foods included in the fibre enrichment intervention	Foods excluded from the fibre enrichment intervention
Bakery products including but not limited to bread, rolls, breakfast cereals and biscuits	100 % fruit and vegetable juices, nectars, juice drinks and soft drinks
Flavoured dairy products such as yogurt drinks	Coffee, tea and infusions
Baked confectionery	Sugar confectionery
Canned soups and powdered soups and sauces to be reconstituted	Foods considered a traditional commodity such as milk, grains or cheese
Some beverages which could reasonably be enriched with fibre, such as fruit smoothies and malt-based powdered beverage drinks	Composite dishes (foods containing several ingredients) with a wide variety of ingredients unclear whether eligible for fibre enrichment

### Creation of distributions

Market shares were considered by assigning a probability of a food with altered fibre content being consumed. For this study, a simple market share of 50 % was used, meaning that 50 % of the foods of interest consist of an altered fibre product. This was done via the creation of a distribution, with the altered fibre concentrations being assigned a market share of 50 % and therefore a probability of being eaten 50 % of the time for each individual eating event. The original concentrations of fibre had an equal chance, or 50 % probability, of being eaten for each individual eating event also. To ensure that the variability of distributions was considered, multiple iterations of each assessment were conducted so that each eating event was simulated several times. In addition to total population and food consumers (people who consume at least one food or beverage from a given food group), stratification of the population was done by age for closer examination of population subsets, as presented in adult cohort characteristics (Table 2).

**Table 2.** National Diet and Nutrition Survey 2015–2016 adult cohort (≥18) characteristics (Numbers; minimum and maximum values)

			Age		BMI		
Ethnic group	Sex	*n*	Min	Max	Min	Max	Mean
White	All	1353	18	94	16	52·9	27·55
	Female	785	18	94	16	52·9	27·35
	Male	568	18	92	16	47·1	27·82
Black or Black British	All	37	18	83	18	41·6	28·68
	Female	21	22	56	18	41·6	30·76
	Male	16	18	83	19	34·2	26·08
Asian or Asian British	All	58	18	87	17	36·7	26·32
	Female	31	18	64	19	35·3	26·4
	Male	27	18	87	17	36·7	26·23
Mixed ethnic group	All	15	18	72	20	32·4	24·52
	Female	12	18	72	20	32·4	25·06
	Male	3	18	29	22	22·8	22·35
Any other group	All	18	20	67	18	36	27·54
	Female	6	26	67	18	29·4	23·07
	Male	12	20	53	21	36	29·78
Don’t know		5	50	81	24	37	30·07
	Female	3	50	81	26	34·5	29·65
	Male	2	50	78	24	37	30·7
Item not applicable	All	1	62	62	29	28·7	28·67
	Female	1	62	62	29	28·7	28·67
Refuse to say	All	1	52	52	29	29·4	29·44
	Male	1	52	52	29	29·4	29·44
Total		1488	18	94	16	52·9	27·51

### Dietary reference values

The number of subjects within the population that meet dietary reference values (DRV) was quantified at baseline and intervention as they were likely to change. DRV for AOAC fibre were obtained from the 2015 Scientific Advisory Committee on Nutrition report on carbohydrates and health^(10)^ as follows: children 2–5 years of age have a DRV of 15 g fibre/d, children aged 6–10 years 20 g fibre/d, children aged 11–16 years 25 g fibre/d and children aged 17 and up along with adults 30 g fibre/d. The percentage of the population meeting the DRV for fibre was calculated and the results were compared at baseline and intervention, with the full cohort broken down into age-based subpopulations. The age-based subpopulations were defined by the Scientific Advisory Committee on Nutrition. There were no missing data, and the full cohort data were used for the modelling (*n* 2723).

### Health outcomes

Understanding the likely impact of reformulation on health outcomes is likely to be one of the key drivers for its adaptation. Analysis using health outcome-based algorithms is a means to express the effects of the reformulation project in terms of a real-world impact. This was done using algorithms sourced from literature which were applied to the baseline intake and intervention intake results for fibre. An algorithm linked to the health outcome was applied to intake results, on the condition that the subject met the criteria detailed. If there was a missing variable required for the modelling of the health outcomes, the subject was excluded.

### Fibre enrichment health outcomes

#### Weight reduction

Following the methodology described by Reynolds *et al.*,^(2)^ subjects were categorised into low (0–25 g/d) and high (≥25 g/d) intakes at baseline and intervention. Where individuals moved from low intake at baseline to high intake at intervention, a 0·37-kg body weight reduction was applied. Only subjects aged 18 years or older were included in the weight change modelling (*n* 1488), as per the Reynolds *et al.* methodology.

#### CVD risk

Estimating the effect of fibre enrichment on cardiovascular health was a multistep process. A CVD risk value was calculated using the algorithm sourced from D’Agostino *et al.* ^(11)^ Antihypertensive medication use was determined by the physician or self-report. If subjects’ blood pressure medication, insulin or oral hypoglycaemic medications were not recorded, it was assumed the subject was not on these medications. Where the subject’s diabetic status was not recorded, it was assumed the subject was not diabetic. Subjects aged 18 years or older, with recorded NDNS data on LDL-cholesterol, total cholesterol, A1C (also known as HbA1C test, a common test used to diagnose diabetes, recorded in the NDNS), systolic blood pressure and smoking status were all included in the CVD risk modelling (*n* 602). A polynomial regression was fitted using data from Reynolds *et al.* ^(2)^ from which a cardiovascular risk reduction value was calculated for each subject. A polynomial regression was used here as the data were non-linear. The reduction was applied to subjects which achieved an increase in fibre consumption at intervention from baseline and consumed more than 15 g of fibre and less than 35 g of fibre at intervention.

#### Risk of type 2 diabetes

The QDiabetes algorithm^(12)^ was used as the first part of a two-step process to estimate the effect of fibre enrichment on type 2 diabetes risk. A simple linear regression was fitted using data from Reynolds *et al.* ^(2)^ A type 2 diabetes risk reduction score was calculated using the line and applied to subjects which achieved an increase in fibre consumption at intervention and consumed more than 15 g fibre and less than 35 g of fibre at intervention. Subjects aged 25–84 years with recorded height and body weight data were included in the type 2 diabetes modelling (*n* 1183), as per the QDiabetes algorithm.

### Data availability

The algorithms used for the health outcomes aspect of this study are available from their respective authors. The data used from the NDNS are available upon request to the UK Data Archive^(4)^. The probabilistic model used to calculate the exposure estimates is the Food Data Science model on Expert Models, available upon licence from Creme Global^(5,6)^.

## Results

### Fibre baseline intakes

The results of the baseline intake assessment showed that the overall mean fibre intake ranged from 11·6 to 18·6 g/d depending on age group (Table 3). The full results of the baseline intakes can be found in online Supplementary Material A: Full Results of Baseline and Intervention Assessments. At baseline, 14·9 % of children aged 2–5 years were achieving the DRV of 15 g/d or more of fibre, 10·6 % of children aged 6–10 years were achieving the DRV of 20 g/d and only 5·7 % of children aged 11–16 years were achieving the DRV of 25 g fibre/day (Table 4). Only 8 % of subjects aged 17+ years were achieving the DRV of 30 g/d or more of fibre (Table 4).

**Table 3.** Fibre baseline and intervention intakes (g/d) and percentage change* (Numbers; mean values and percentages)

		Baseline (g fibre/d)		Intervention (g fibre/d)		% Change in fibre intake	
Age (years)	*n*	Mean	P95	Mean	P95	Mean	P95
2–5	365	11·6	17·2	13·6	20	+14·7	+14
6–10	359	14·5	22·1	16·8	25·3	+13·7	+12·6
11–16	387	15·2	25·3	17·5	28·7	+13·1	+11·8
17–94	1572	18·6	32·2	20·8	35·7	+10·6	+9·8

* National Diet and Nutrition Survey sampling weights have been applied to all estimated results.

**Table 4.** Population meeting fibre DRV at baseline and intervention* (Numbers and percentages)

Age (years)	*n*	% meeting DRV at baseline	% meeting DRV at intervention	% change
2–5	365	14·9	32·5	+218·1
6–10	359	10·6	22·4	+211·3
11–16	387	5·7	9·4	+164·9
17–94	1572	8·0	12·2	+52·5

DRV, dietary reference value.

* National Diet and Nutrition Survey sampling weights have been applied to all estimated results.

### Fibre intervention intake assessments

The results of the fibre enrichment intervention scenario showed that the overall mean fibre intake in the UK post-intervention ranged from 13·6 to 20·8 g/d depending on age, with all age groups consuming more fibre compared with the baseline diet (Table 3). Children aged 0–17 years achieved a 2·1 g/d fibre intake increase from their baseline diet, with a mean intake of 16·1 g/d after intervention (Table 3). At intervention, the percentage of children aged 2–5 years and 6–10 years achieving the DRV of fibre more than doubled in comparison with the baseline assessment, at 32·5 and 22·4 %, respectively (Table 4). The percentage of children aged 11–16 years achieving the DRV rose from 5·7 % at baseline to 9·4 % at intervention (Table 4). Adults achieving the DRV of 30 g/d or more of fibre rose from 8·0 % at baseline to 12·2 % at intervention. Adults and older children (17+) achieved a 12·1 % fibre intake increase from their baseline diet, with a mean intake of 20·8 g/d after intervention. The full results of the baseline and intervention intakes can be found in online Supplemental Material A: Full Results of Baseline and Intervention Assessments.

### Health outcomes

The results below show summary statistics for health outcome results. Wilcoxon signed-rank test was conducted to test the null hypothesis that two related paired samples come from the same distribution. The result was found to be statistically significant if *P* ≤ 0·05. A Wilcoxon signed-rank test was applied here as the data were not normally distributed. In the context of this study, a ‘paired sample’ refers to the same subject pre- and post-intervention, or pre- and post-fibre enrichment.

### Fibre consumption and weight change

A modest change was observed between baseline and intervention for fibre consumption and weight change. A mean body weight of 70·36 kg observed at baseline was reduced by 0·03 kg at intervention to a mean body weight of 70·33 kg with 5·9 % of subjects achieving a weight reduction. Wilcoxon signed-rank test showed the results were statistically significant (*P* ≤ 0·05).

#### Fibre consumption and cardiovascular risk

**Fig. 1.**
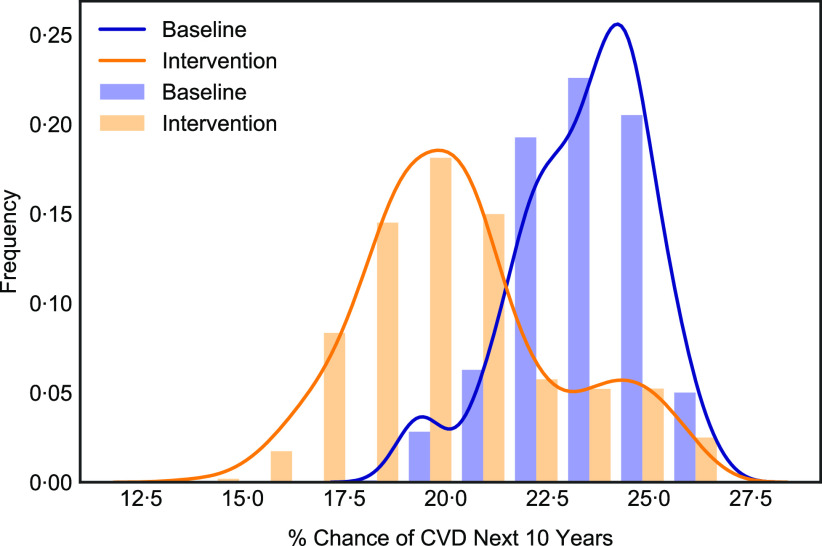
CVD risk (10-year percentage chance of CVD) distribution plot. Fibre enrichment ‘Baseline’ scenario in blue and ‘Intervention’ in orange (*n* 602).

The CVD risk distribution curve shown in Fig. 1 shifted 13 % to the left towards lower CVD risk over the next 10 years post-fibre intervention, with the most frequent risk value being approximately 20 % at intervention compared with approximately 24 % at baseline. It was observed that 72·2 % of subjects achieved a reduction in cardiovascular risk. Wilcoxon signed-rank test showed the results were statistically significant (*P* ≤ 0·05). Post-intervention, the greatest reductions in CVD risk were between the 5th and 75th percentiles, compared with baseline. The bar chart shows 75 % of people with a CVD risk value of approximately 23 % or less experienced the greatest benefit from fibre enrichment in the diet post-intervention.

#### Fibre consumption and type 2 diabetes

**Fig. 2.**
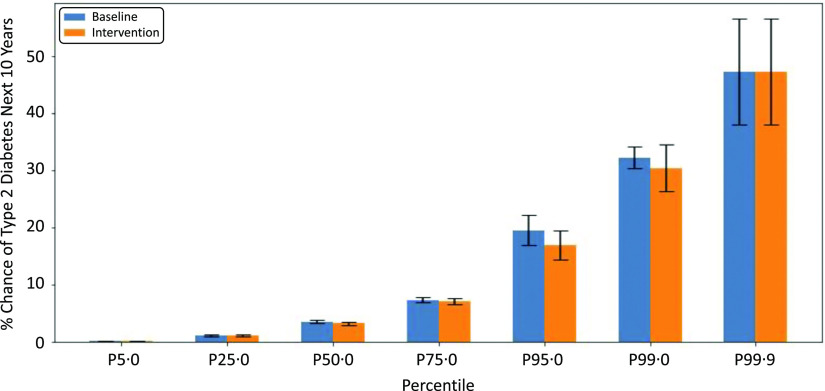
Type 2 diabetes risk (10-year percentage chance) percentiles. Fibre enrichment ‘Intervention’ scenario in blue and ‘Baseline’ in orange. The greatest change between baseline and intervention is observed at the higher percentiles, indicating those most at risk at baseline will benefit the most from fibre enrichment in terms of diabetes risk in the post-intervention scenario (*n* 1183).

A reduction in type 2 diabetes risk was observed post-intervention in the fibre enrichment scenario as seen in the higher peaks at lower type 2 diabetes risk values (Fig. 2). A mean of a 5·45 % chance of developing type 2 diabetes within the next 10 years at baseline was reduced to 4·98 % at intervention. A reduction in type 2 diabetes risk was observed in 71·7 % of subjects. Wilcoxon signed-rank test showed the results were statistically significant (*P* ≤ 0·05).

## Discussion

Fibre is gaining more attention of late due to benefits beyond its traditional support of laxation around regular bowel movements. While decades of evidence have demonstrated the benefits of fibre on cardiovascular and metabolic health, there is emerging evidence on fibre’s benefits on immune, brain and skin health^(2)^. Understanding the likely public health impacts of increased fibre consumption may serve as an encouragement for food and beverage manufacturers to reformulate and help policy makers to develop supporting frameworks.

Unsurprisingly, the majority of UK adults were still not consuming the recommended values of fibre at the modelled intervention. However, the relative increases measured using the DRV and algorithms showed the strong impact a 50 % level of reformulation can have on the population. In the present study, at baseline the UK population mean fibre intake from all foods ranged from 11·6 to 18·6 g/d depending on age group. The fibre enrichment intervention scenario showed that the overall mean fibre intake post-intervention ranged from 13·6 to 20·8 g/d depending on age, with all age groups consuming more fibre compared with the baseline diet. To put this into context, fibre enrichment would result in the number of children under 10 years old consuming the recommended amount of fibre daily more than double and the number of adults meeting the DRV rise by 53 %. In a similar dietary modelling study, Nicklas *et al.* looked at adding fibre to grain products low in dietary fibre at a level of 2·5 or 5·0 g per serving, which was the US FDA ‘good’ and ‘excellent’ source levels at the time of publication^(13)^. This level of fortification resulted in 24·7 and 39·1 g of fibre consumed a day, respectively, based on American eating patterns as observed in the U.S. National Health and Nutrition Examination Survey (NHANES) database and fortification of all grain products. This publication reported higher level of fibre intakes per day due to the fortification of all grain products, whereas our study was only evaluating 50 % market share.

These positive results of increased fibre intake were reinforced by the use of algorithms, which predicted risk reductions of type 2 diabetes and CVD, as well as body weight reduction. Per the British Heart Foundation, in 2019, there were 168 472 deaths from ‘all heart and circulatory diseases’^(14)^, or approximately 461 per day. A potential 13 % reduction in cardiovascular deaths would have led to a reduction of 21 902 deaths in 2019 or approximately 60 deaths per day. Total deaths from CVD could have dropped to 146 570 post-intervention as suggested in the fibre enrichment scenario. To provide the scale of impact on diabetes, we used Diabetes UK-published figures based on newly diagnosed cases of diabetes from the 2011/2012 and 2012/2013 National Diabetes Audit^(15)^. The data state that approximately 700 people a day are diagnosed with diabetes^(14)^, with approximately 90 % of these cases being type 2 diabetes. Since a 8·6 % reduction (5·45 % baseline to 4·98 % post-hypothetical intervention) in type 2 diabetes risk was observed in the fibre enrichment modelling scenario, on average for the selected population, this would translate into a reduction of fifty-four type 2 diabetes cases per day or 19 710 per annum with this level of proposed fibre reformulation.

In Reynolds *et al.* systematic reviews and meta-analyses, it was estimated that the greatest risk reductions associated with a range of critical outcomes were achieved when daily dietary fibre intakes were between 25 and 29 g^(2)^. The current modelling study did not achieve these levels of adult fibre intakes due to our estimation of a 50 % market share of fibre addition at moderate amounts of 3 g per 100 g for foods, adding 3 g of fibre to foods which had 3 g already or 1·5 g per 100 kcal of beverages. In this meta-analysis of higher fibre levels, there was a correlation of six fewer cases of CHD per 1000 participants which cannot be well compared with our modelling of CVD.

This study gives a good overview of the potential public health benefits of reformulating food products using a straightforward enrichment scenario. Going forward, this scenario can be expanded to include more varied reformulation methods and to incorporate complementary data (such as a more refined market share) to improve the realism of results. Understanding the likely public health impact of fibre addition may serve as an encouragement for food and beverage manufacturers to reformulate and help policy makers to develop a supporting framework to do so. The UK NDNS allowed such analysis because of the granularity of the data and consistent methodologies.

A formal uncertainty analysis was not conducted as part of the study; however, the authors acknowledge that there are some limitations of this modelling study which introduce uncertainty into the conclusion. These are data for the UK population, and these results may not be representative of other countries with different dietary profiles. Therefore, the model may need to be refined for other countries based on the availability of data for dietary patterns as well as availability of nutrition composition tables of products. This was an approach of adding fibre to appropriate products at a level of 50 % market share and the fibre increase values as well as the health impacts are based on this assumed level of reformulation. These results of public health impact are based on different published algorithms with varying specificity and sensitivity. For example, while results were statistically significant for weight reduction, the 0·03-kg weight change with fibre reformulation might not be meaningful in terms of a physiological public health outcome. An assumption made in this publication is that since the EU Commission Directive 2008/100/EC Annex II, FDA in 21CFR §101·9 (e) (6) (i), *Codex Alimentarius* and other regulatory authorities approved fibres based on health benefits, it is presumed in this publication’s modelling that human health benefits will extend to these novel synthesised or extracted fibres as well. Comparing and contrasting the human health benefits of intrinsic fibres found in whole grains, legumes, fruits and vegetables to fibres extracted or synthesised from those sources would be fascinating and a needed addition to our understanding of fibre’s impact on human health. Another limitation of the study is a lack of quantification of potential adverse or unintended consequences of adding fibre to foods and beverages. The potential impact of how fortification may shift consumption patterns of different food groups, in particular that of fresh and packaged foods, should be the focus for future research. Based on the modelling from this study, there should not be an excessive quantity of fibre consumed by any age groups.

Fibre was originally thought of as a nutrient for digestive health with laxation and regularity; yet with greater understanding of the colonic microbiota, fibre fermentation and production of SCFA, an improved understanding of how fibre impacts multiple organ systems is emerging. Fibres may have the ability to affect inflammation, metabolism, carcinogenesis and immunity pathways positively impacting colon cancer, obesity, CVD and type 2 diabetes risks^(16)^. The value of using health-based algorithms was evident here, as they can be refined to match specific subject data as well as being combined with other algorithms to measure the effects of different health outcomes on the individual and the population.

## Supplementary material

For supplementary material accompanying this paper visit https://doi.org/10.1017/S0007114521004827. click here to view supplementary material
