# Supplementary material for: Estimating the potential public health impact of fibre enrichment: a UK modelling study
Source: Br J Nutr. 2022 Jan 7;128(9):1868–74. doi: 10.1017/S0007114521004827 (PMC9597481; doi:10.1017/S0007114521004827)
Supplement: Supplementary file 1 [file S0007114521004827sup001.docx]

**Supplemental Material A:** **Full Results of Baseline and Fibre Enrichment Intervention Assessments**

|  |  | | *Subpopulation* | | | | | | | | | |
| --- | --- | --- | --- | --- | --- | --- | --- | --- | --- | --- | --- | --- |
|  |  | | 2-5 Years Old | | 6-10 Years Old | | 11-16 Years Old | | 17+ Years Old | | All Ages | |
| *Food Category* |  |  | *Consumers* | *Total Population* | *Consumers* | *Total Population* | *Consumers* | *Total Population* | *Consumers* | *Total Population* | *Consumers* | *Total Population* |
| Beverages | Baseline | ***n*** | 365 | 365 | 359 | 359 | 386 | 387 | 1572 | 1572 | 2722 | 2723 |
|  |  | ***Mean*** | 0.1 | 0.1 | 0.2 | 0.2 | 0.2 | 0.2 | 0.2 | 0.2 | 0.2 | 0.2 |
|  |  | ***P95*** | 0.6 | 0.6 | 1.2 | 1.2 | 0.8 | 0.8 | 0.8 | 0.8 | 0.8 | 0.8 |
|  | Intervention | ***n*** | 365 | 365 | 359 | 359 | 386 | 387 | 1572 | 1572 | 2722 | 2723 |
|  |  | ***Mean*** | 0.1 | 0.1 | 0.3 | 0.3 | 0.2 | 0.2 | 0.2 | 0.2 | 0.2 | 0.2 |
|  |  | ***P95*** | 0.7 | 0.7 | 1.3 | 1.3 | 1.0 | 1.0 | 1.0 | 1.0 | 1.0 | 1.0 |
| % Increase Mean Fibre | |  | **33.9** | **33.9** | **27.9** | **27.9** | **23.5** | **23.5** | **19.8** | **19.8** | **0.0** | **0.0** |
| Bakery | Baseline | ***n*** | 365 | 365 | 359 | 359 | 387 | 387 | 1568 | 1572 | 2719 | 2723 |
|  |  | ***Mean*** | 4.9 | 4.9 | 6.3 | 6.3 | 6.7 | 6.7 | 7.0 | 6.9 | 6.8 | 6.8 |
|  |  | ***P95*** | 8.6 | 8.6 | 11.0 | 11.0 | 11.8 | 11.8 | 13.8 | 13.8 | 13.3 | 13.3 |
|  | Intervention | ***n*** | 365 | 365 | 359 | 359 | 387 | 387 | 1568 | 1572 | 38066 | 38122 |
|  |  | ***Mean*** | 6.0 | 6.0 | 7.8 | 7.8 | 8.3 | 8.3 | 8.5 | 8.5 | 8.3 | 8.3 |
|  |  | ***P95*** | 10.8 | 10.8 | 13.8 | 13.8 | 14.9 | 14.9 | 16.9 | 16.9 | 16.4 | 16.4 |
| % Increase Mean Fibre | |  | **22.6** | **22.6** | **23.7** | **23.7** | **23.0** | **23.0** | **21.9** | **21.9** | **22.1** | **22.1** |
| Dairy and Dairy Alternatives | Baseline | ***n*** | 365 | 365 | 357 | 359 | 381 | 387 | 1551 | 1572 | 2694 | 2723 |
|  |  | ***Mean*** | 0.2 | 0.2 | 0.1 | 0.1 | 0.1 | 0.1 | 0.1 | 0.1 | 0.1 | 0.1 |
|  |  | ***P95*** | 0.5 | 0.5 | 0.4 | 0.4 | 0.8 | 0.7 | 0.6 | 0.6 | 0.6 | 0.6 |
|  | Intervention | ***n*** | 365 | 365 | 357 | 359 | 381 | 387 | 1551 | 1572 | 2694 | 2723 |
|  |  | ***Mean*** | 0.6 | 0.6 | 0.5 | 0.5 | 0.3 | 0.3 | 0.3 | 0.3 | 0.4 | 0.4 |
|  |  | ***P95*** | 2.3 | 2.3 | 2.1 | 2.0 | 1.9 | 1.9 | 1.9 | 1.9 | 1.9 | 1.9 |
| % Increase Mean Fibre | |  | **251.8** | **251.8** | **410.7** | **410.7** | **159.4** | **159.4** | **188.5** | **188.5** | **300.0** | **300.0** |

P95, 95^th^ percentile

**Supplemental Material A continued**

|  | | | *Subpopulation* | | | | | | | | | |
| --- | --- | --- | --- | --- | --- | --- | --- | --- | --- | --- | --- | --- |
|  |  |  | 2-5 Years Old | | 6-10 Years Old | | 11-16 Years Old | | 17+ Years Old | | All Ages | |
| *Food Category* |  | | *Consumers* | *Total Population* | *Consumers* | *Total Population* | *Consumers* | *Total Population* | *Consumers* | *Total Population* | *Consumers* | *Total Population* |
| Soups, Sauces and Dressings | Baseline | ***n*** | 311 | 365 | 309 | 359 | 351 | 387 | 1408 | 1572 | 2412 | 2723 |
|  |  | ***Mean*** | 0.2 | 0.2 | 0.2 | 0.2 | 0.3 | 0.3 | 0.4 | 0.3 | 0.4 | 0.3 |
|  |  | ***P95*** | 0.6 | 0.6 | 0.6 | 0.6 | 1.0 | 0.9 | 1.3 | 1.2 | 1.2 | 1.2 |
|  | Intervention | ***n*** | 311 | 365 | 309 | 359 | 351 | 387 | 1408 | 1572 | 2412 | 2723 |
|  |  | ***Mean*** | 0.4 | 0.3 | 0.5 | 0.4 | 0.7 | 0.6 | 0.8 | 0.7 | 0.7 | 0.6 |
|  |  | ***P95*** | 1.4 | 1.3 | 1.5 | 1.3 | 2.3 | 2.2 | 3.0 | 2.7 | 2.7 | 2.5 |
| % Increase Mean Fibre | |  | **99.4** | **99.4** | **101.2** | **101.2** | **96.0** | **96.0** | **96.4** | **96.4** | **75.0** | **100.0** |
| Confectionery | Baseline | ***n*** | 345 | 365 | 350 | 359 | 363 | 387 | 1433 | 1572 | 2522 | 2723 |
|  |  | ***Mean*** | 0.3 | 0.3 | 0.4 | 0.4 | 0.5 | 0.5 | 0.4 | 0.3 | 0.4 | 0.4 |
|  |  | ***P95*** | 1.0 | 0.9 | 1.3 | 1.3 | 2.0 | 1.8 | 1.3 | 1.3 | 1.3 | 1.3 |
|  | Intervention | ***n*** | 345 | 365 | 350 | 359 | 363 | 387 | 1433 | 1572 | 2522 | 2723 |
|  |  | ***Mean*** | 0.5 | 0.5 | 0.6 | 0.6 | 0.7 | 0.6 | 0.5 | 0.5 | 0.5 | 0.5 |
|  |  | ***P95*** | 1.7 | 1.7 | 1.8 | 1.8 | 2.6 | 2.5 | 2.0 | 2.0 | 2.0 | 2.0 |
| % Increase Mean Fibre | |  | **60.7** | **60.7** | **42.1** | **42.1** | **35.7** | **35.7** | **43.9** | **43.9** | **25.0** | **25.0** |
| Other | Baseline | ***n*** | 365 | 365 | 359 | 359 | 387 | 387 | 1572 | 1572 | 2723 | 2723 |
|  |  | ***Mean*** | 6.0 | 6.0 | 7.2 | 7.2 | 7.4 | 7.4 | 10.7 | 10.7 | 10.0 | 10.0 |
|  |  | ***P95*** | 10.9 | 10.9 | 12.7 | 12.7 | 13.9 | 13.9 | 21.5 | 21.5 | 20.3 | 20.3 |
|  | Intervention | ***n*** | 365 | 365 | 359 | 359 | 387 | 387 | 1572 | 1572 | 2723 | 2723 |
|  |  | ***Mean*** | 6.0 | 6.0 | 7.2 | 7.2 | 7.4 | 7.4 | 10.7 | 10.7 | 10.0 | 10.0 |
|  |  | ***P95*** | 10.9 | 10.9 | 12.8 | 12.8 | 13.9 | 13.9 | 21.6 | 21.6 | 20.3 | 20.3 |
| % Increase Mean Fibre | |  | **0.0** | **0.0** | **0.0** | **0.0** | **0.0** | **0.0** | **0.0** | **0.0** | **0.0** | **0.0** |
| Total Diet | Baseline | **n** | 365 | 365 | 359 | 359 | 387 | 387 | 1572 | 1572 | 2723 | 2723 |
|  |  | **Mean** | 11.6 | 11.6 | 14.5 | 14.5 | 15.2 | 15.2 | 18.6 | 18.6 | 17.7 | 17.7 |
|  |  | **P95** | 17.2 | 17.2 | 22.1 | 22.1 | 25.3 | 25.3 | 32.2 | 32.2 | 31.4 | 31.4 |
|  | Intervention | **n** | 365 | 365 | 359 | 359 | 387 | 387 | 1572 | 1572 | 2723 | 2723 |
|  |  | **Mean** | 13.6 | 13.6 | 16.8 | 16.8 | 17.5 | 17.5 | 20.8 | 20.8 | 19.9 | 19.9 |
|  |  | **P95** | 20.0 | 20.0 | 25.3 | 25.3 | 28.7 | 28.7 | 35.7 | 35.7 | 34.7 | 34.7 |
| % Increase Mean Fibre | |  | **16.6** | **16.6** | **16.0** | **16.0** | **14.9** | **14.9** | **12.1** | **12.1** | **12.4** | **12.4** |

P95, 95^th^ percentile
